# Complete genome sequence of “Bacillaceae sp. strain IKA-2”: a cold-active, amylase-producing bacterium from ikaite columns in SW Greenland

**DOI:** 10.1128/mra.00887-23

**Published:** 2023-12-22

**Authors:** A. Zervas, P. Stougaard, M. S. Thøgersen

**Affiliations:** 1 Department of Environmental Science, Aarhus University, Roskilde, Denmark; California State University San Marcos, San Marcos, California, USA

**Keywords:** genome, cold-active, bacterium, Bacillaceae, Greenland

## Abstract

Bacillaceae sp. strain IKA-2 is a bacterium isolated from the permanently cold and alkaline ikaite columns in the Ikka Fjord in SW Greenland (61°12′05″N; 48°00′50″W). The bacterium grows well at 10°C in a substrate buffered to pH 10. It has a genome size of 4,424,890 bp and a guanine-cytosine (GC) content of 36.2%. The genome harbors genes involved in hydrolysis of long carbohydrates and in protection against cold shock.

## ANNOUNCEMENT

The ikaite columns in SW Greenland are a unique environment since it is the only known place on Earth that is permanently cold (<10°C) with a high pH (10.4) (1, 2). Bacteria from this environment have previously been shown to produce cold-active enzymes with industrial relevance (3–6). Three ikaite columns were collected during the summer of 2019 (license no. G19-032), cut into pieces of 20–50 cm each, and stored at −20°C for transportation back to the laboratory. One of these ikaite pieces was used for the isolation of enzyme-producing bacteria. The cut surface was removed to avoid “contamination” from handling and contact with seawater, and the material was drilled from the interior of the column. When drilled, the ikaite material, due to its metastable structure, disintegrates into a sand-like slurry. This slurry was used to inoculate agar plates containing substrates to select for specific enzymatic activities. During a screening campaign for novel hydrolytic enzymes, the ikaite material was plated onto 1/10 diluted R2 agar medium without glucose buffered to pH 10 (50 mM NaHCO_3_/Na_2_CO_3_) and supplemented with 0.05 g/L soluble starch and 0.5 g/L AZCL-amylose (Megazyme, Wicklow, Ireland). The plates were incubated at 5°C for 75 days. A single colony showing hydrolytic activity by solubilizing the azurine-crosslinked (AZCL)-amylose substrate was isolated and initially identified by 16S rRNA gene sequencing. As the 16S rRNA gene sequence indicated a new bacterial species, the strain was selected for whole genome sequencing.

**Fig 1:**
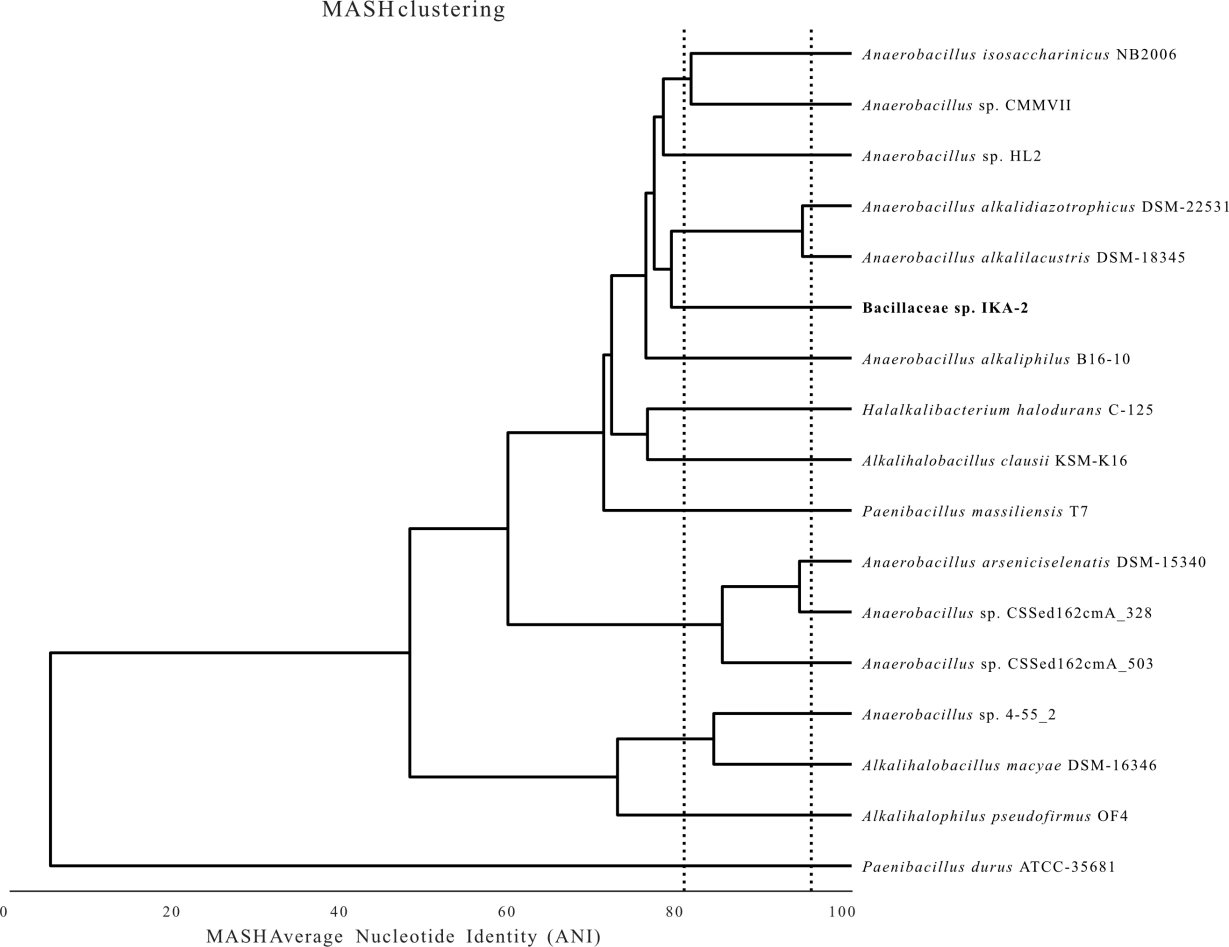
Distance tree based on whole genome sequences of Bacillaceae sp. strain IKA-2 (this study) and the complete genomes of the closest related species based on 16S sequence similarity. The tree was reconstructed using dRep according to its “compare” algorithm. The dotted lines indicate 80% and 95% identity, accepted generally as species and genus delimitation thresholds, respectively.

For isolation of high molecular weight DNA, a single colony was used for inoculation of R2 broth at pH 10 (as above) and incubated at 10°C, 200 rpm for 6 days. DNA was extracted from 2 × 25 mL culture using the MasterPure Complete DNA & RNA Purification kit (Lucigen, Middleton, WI, USA). Illumina libraries were prepared using the Nextera XT library preparation kit (Illumina, San Diego, CA, USA) following the manufacturer’s instructions and sequenced on a NextSeq 500 using the 300 cycles v.2.5 chemistry (151 bp pair-end sequencing). From the same DNA, Oxford Nanopore Technologies libraries were prepared using the Ligation Sequencing kit v.14 (SQK-LSK114), without size selection, and loaded on a MinION using an R10 flowcell. All programs mentioned were run with default settings unless otherwise stated. The run was controlled by MinKnow 23.04.5, and basecalling was done by Guppy version 6.5.7+ca6d6af. In total, we generated 5,452,536 raw, pair-end Illumina reads and 655,629 raw Oxford Nanopore Technologies (ONT) reads. Average read length and N50 of the ONT reads were 2,394.6 bp and 4,038, respectively, as calculated by NanoStat v.1.6.0 (7). Adapters were trimmed from the long reads using Porechop v.0.2.4 (https://github.com/rrwick/Porechop). Raw Illumina reads were subjected to quality control and contamination removal using trim-galore v.0.6.5 (https://github.com/FelixKrueger/TrimGalore) and were used to further improve the Nanopore reads with LorDEC v.0.9 (8), which were used for *de novo* whole genome assembly with Flye v.2.9-b1768 (9) utilizing the –nano-corr. The assembly graph was visualized using Bandage v.0.8.1 (10), while the quality of the genome was verified using BUSCO v.5.4.0 (11) and its generalized bacteria odb10 (2020-03-06) database. The complete genome was annotated using the NCBI prokaryotic genome annotation pipeline (12) (done by NCBI during submission of the genome). It consists of a chromosome (4,424,890 bp) with a G + C content of 36.2% and does not contain plasmids. In total, 4,292 genes were predicted on the genome including 87 tRNA, 1 tmRNA (transfer-messenger RNA), 29 rRNA in 10 operons, 4 ncRNA, and 4,171 protein-coding sequences. The annotated 16S genes were used for BLAST searches (blastn) against NCBI’s 16S database, implemented in Geneious Prime 2023.2.1 (Biomatters). The closest related sequences with taxonomic information belonged to the genera *Anaerobacillus*, *Paenibacillus*, *Alkalihalobacillus*, and *Halalkalibacterium* (sequence similarity <96.7%). We compared average nucleotide identities between the IKA-2 strain and 13 genomes belonging to these genera using dRep v.3.4.0 (13). The resulting distance tree showed Average Nucleotide Identity (ANI) similarity <80% between IKA-2 and the available, closely related genomes (Fig. 1). Thus, IKA-2 cannot be assigned to a specific genus, and it most likely represents a novel taxon within Bacillaceae.

## Data Availability

Raw Illumina pair-end reads and raw Oxford Nanopore Technologies long reads were deposited in NCBI at the SRA and are available under the accession numbers SRX21753929 and SRX21753930, respectively. The complete genome sequence of IKA-2 is available at NCBI under accession number CP134492.
